# Observation of momentum-resolved charge fluctuations proximate to the charge-order phase using resonant inelastic x-ray scattering

**DOI:** 10.1038/srep23611

**Published:** 2016-03-29

**Authors:** M. Yoshida, K. Ishii, M. Naka, S. Ishihara, I. Jarrige, K. Ikeuchi, Y. Murakami, K. Kudo, Y. Koike, T. Nagata, Y. Fukada, N. Ikeda, J. Mizuki

**Affiliations:** 1 SPring-8, Japan Atomic Energy Agency, Sayo, Hyogo 679-5148, Japan; 2 Department of Physics, Graduate School of Science, Tohoku University, Sendai 980-8578, Japan; 3 RIKEN Center for Emergent Matter Science (CEMS), Wako 351-0198, Japan; 4 CREST, JST, Chiyoda, Tokyo 102-0076, Japan; 5 Research Center for Neutron Science and Technology, Comprehensive Research Organization for Science and Society (CROSS), Tokai, Ibaraki 319-1106, Japan; 6 Institute of Materials Structure Science, High Energy Accelerator Research Organization, Tsukuba, Ibaraki 305-0801, Japan; 7 Department of Physics, Okayama University, Okayama 700-8530, Japan; 8 Department of Applied Physics, Graduate School of Engineering, Tohoku University, Sendai 980-8579, Japan; 9 School of Science and Technology, Kwansei Gakuin University, Sanda, Hyogo 669-1337, Japan

## Abstract

In strongly correlated electron systems, enhanced fluctuations in the proximity of the ordered states of electronic degrees of freedom often induce anomalous electronic properties such as unconventional superconductivity. While spin fluctuations in the energy-momentum space have been studied widely using inelastic neutron scattering, other degrees of freedom, i.e., charge and orbital, have hardly been explored thus far. Here, we use resonant inelastic x-ray scattering to observe charge fluctuations proximate to the charge-order phase in transition metal oxides. In the two-leg ladder of Sr_14−*x*_Ca_*x*_Cu_24_O_41_, charge fluctuations are enhanced at the propagation vector of the charge order (***q***_CO_) when the order is melted by raising temperature or by doping holes. In contrast, charge fluctuations are observed not only at ***q***_CO_ but also at other momenta in a geometrically frustrated triangular bilayer lattice of LuFe_2_O_4_. The observed charge fluctuations have a high energy (~1 eV), suggesting that the Coulomb repulsion between electrons plays an important role in the formation of the charge order.

Strongly correlated electron systems show many interesting and important properties such as high Tc superconductivity, colossal magnetoresistive effect, multi-ferroics, and giant thermoelectric conversion. These properties appear in or in the vicinity of the Mott insulating state in which some of the degrees of freedom associated with the electron are ordered, and these are commonly believed to be driven by the strong Coulomb interaction. In these systems, enhanced fluctuations of spin, charge, and orbital correlations in the proximity of the ordered states play an important role for the attractive properties mentioned above. Therefore, spin fluctuations in the energy-momentum space are widely studied by inelastic neutron scattering^1^,^2^,^3^,^4^. Recently, resonant inelastic x-ray scattering (RIXS) at the transition metal *L*-edge has become a complementary technique in measuring spin-flip magnetic excitations^5^,^6^. In contrast, charge fluctuations have hardly been explored in the energy- and momentum-resolved fashion so far because of the limitation of experimental methods. In principle, inelastic scattering of photons or electrons is directly connected to dynamical charge correlation, but these techniques have shortcomings: nonresonant inelastic x-ray scattering usually suffers from low cross-section, and inelastic electron scattering (electron energy loss spectroscopy, EELS) presents difficulties in sample preparation and interpretation due to multiple scattering. Under these circumstances, RIXS at the transition metal *K*-edge, which is referred to as indirect RIXS^7^, is a possible candidate for observing charge fluctuations because resonant enhancement facilitates the observation of charge excitations within a realistic time frame. Up till now, it has been demonstrated that *K*-edge RIXS spectra are qualitatively similar to the dynamical charge correlation function *N*(***q***, *ω*)^8^, and the cross-section of *K*-edge RIXS is represented by *N*(***q***, *ω*) under a certain approximation^9^,^10^.

We apply the *K*-edge RIXS technique to two systems that show a charge order and observe the momentum-resolved charge fluctuations proximate to the charge-ordered phase in strongly correlated transition metal oxides. One is Sr_14−*x*_Ca_*x*_Cu_24_O_41_, which is a composite crystal consisting of a two-leg ladder and an edge-shared chain with different periodicity. The ladder part with a large transfer energy of carriers is responsible for most physical properties. Isovalent substitution of Ca for Sr causes transfer of holes from the chain to the ladder^11^,^12^, and superconductivity at large Ca concentrations under high pressure has attracted great interest^13^,^14^. The nominal valence of Cu is +2.25, and holes are already doped in the ladder in parent Sr_14_Cu_24_O_41_. The charge-order formation at *x* = 0 was established from impedance measurements^15^,^16^, and a resonant elastic x-ray scattering study confirmed a five-fold periodicity of the charge order^17^. When additional holes are doped into the ladder by partially substituting Ca for Sr, the order melts suddenly^18^. The other system is the triangular bilayer in LuFe_2_O_4_, in which equal amounts of Fe^2+^ and Fe^3+^ coexist. A three-fold charge order is realized in LuFe_2_O_4_^19^, but the charge is situated on a frustrated geometry in contrast to the two-leg ladder in Sr_14−*x*_Ca_*x*_Cu_24_O_41_. It has been theoretically argued that the frustration leads to the formation of a novel 3-fold charge order with electric polarization^20^. Note that a recent structural analysis proposed a nonpolar charge arrangement within the bilayer in contrast to the previously believed polar bilayer^21^. Nevertheless, the geometrical charge frustration and the three-fold charge order, which are the two important features in the present study, are maintained.

In the present study, we observe the charge fluctuations in the momentum-energy space induced by thermal or quantum melting of the charge order of strongly correlated electrons. These fluctuations appear as a continuum spectral weight in RIXS spectra and show characteristic momentum dependence: the spectral weight is enhanced only at the propagation vector of the charge order (***q***_CO_) in the two-leg ladder lattice, whereas it is observed at some momentum points in the triangular bilayer lattice. The latter case is analogous to geometrical spin frustration, namely competition among various charge configurations emerges as charge fluctuations which spread across the wide momentum space.

## Results

### Excitations in the two-leg ladder lattice

**Figure 1:**
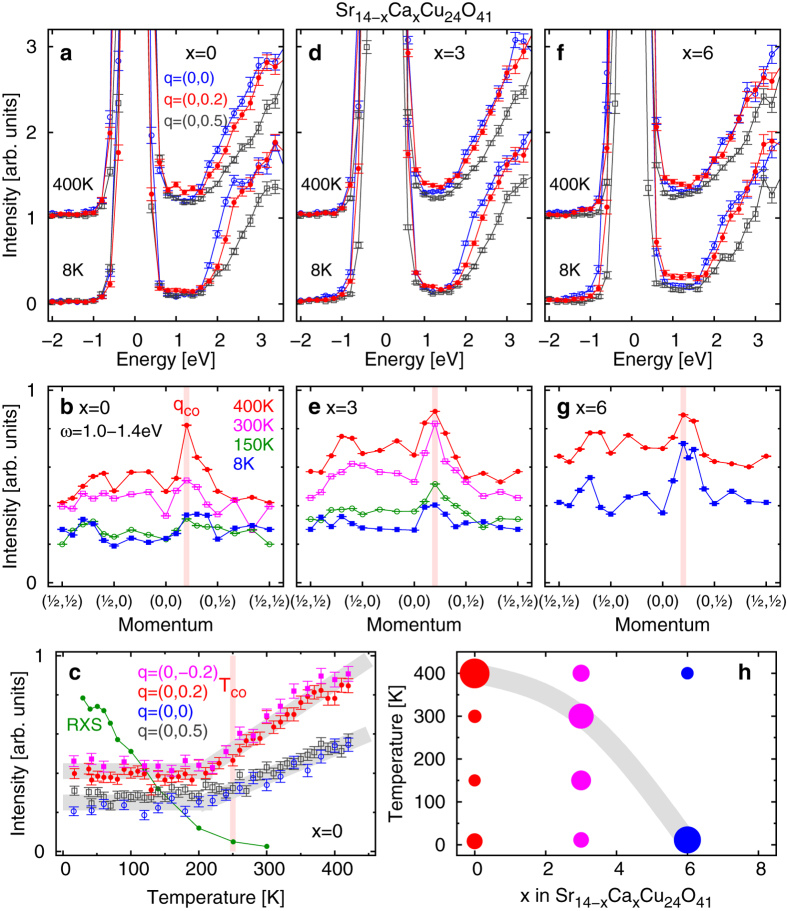
Charge excitations in the two-leg ladder lattice in Sr_14−*x*_Ca_*x*_Cu_24_O_41_. (**a**,**d**,**f**) Raw RIXS spectra of *x* = 0, 3 and 6 measured at 8 K and 400 K. (**b**,**e**,**g**) Momentum dependence of integrated spectral weight at 1.0–1.4 eV. The vertical thick bars denote the propagation vector of the charge order at *x* = 0 (***q***_CO_). (**c**) Temperature dependence of integrated spectral weight at 1.0–1.4 eV. Intensity of resonant elastic x-ray scattering (RXS) taken from ref. 17 is also presented. The vertical thick bar shows the transition temperature of the charge order (*T*_CO_). (**h**) Enhancement of the RIXS intensity at ***q***_CO_. The definition is given in the main text. The thick solid lines in (**c**,**h**) are a guide for eyes.

We begin with the two-leg ladder lattice in Sr_14−*x*_Ca_*x*_Cu_24_O_41_. Figure 1a shows Cu *K*-edge RIXS spectra of the parent Sr_14_Cu_24_O_41_ below (8 K) and above (400 K) the charge-order transition temperature *T*_CO_ ≈ 250 K. Because the momentum dependence perpendicular to the ladder plane is weak, we discuss the reduced momentum in the ladder plane represented as ***q*** = (*q*_rung_, *q*_leg_). The intense signal above 2 eV is ascribed to a charge-transfer excitation from the Zhang-Rice band to the upper Hubbard band of the ladder^22^. It is already observed in an undoped Mott insulating compound. When holes are doped, continuum-like weight appears below the charge-transfer gap and its intensity is proportional to the hole concentration^22^. This spectral weight originates from intraband excitations, namely dynamical response of the doped holes in the ladder. Because the charge order in Sr_14_Cu_24_O_41_ is regarded as a spatial modulation of the doped holes, we can directly investigate the dynamics of doped holes from the intraband excitations around 1 eV. Therefore variation of the spectral weight of the intraband excitations is the main subject of this study.

At 8 K, the momentum dependence of the intensity of the continuum-like spectral weight is weak. Figure 1a shows that it is slightly higher at ***q*** = (0, 0.2) than at the other two momenta, i.e., ***q*** = (0, 0) and (0, 0.5). When the charge order is thermally melted at 400 K, the momentum dependence changes considerably; the intensity is enhanced substantially at ***q*** = (0, 0.2), which coincides with the propagation vector of the five-fold charge order (***q***_CO_). The momentum dependence of the continuum-like spectral weight is shown in Fig. 1b, where we show a plot of the integrated RIXS intensity between 1.0 and 1.4 eV after subtracting the corresponding intensity of the anti-Stokes region (between −1.4 and −1.0 eV). It is clear that the enhanced intensity at 400 K is observed just at ***q***_CO_, while momentum dependence of the intensity is rather flat at lower temperatures. Figure 1c shows the temperature dependence of the integrated RIXS intensity at four momenta, two of which are the propagation vectors of the charge order ***q***_CO_. We superimpose the intensity of resonant elastic x-ray scattering taken from ref. 17, which corresponds to the order parameter of the charge order evolving below the transition temperature (*T*_CO_ ≈ 250 K). While the integrated RIXS intensity is constant at temperatures well below *T*_CO_, it begins to increase with increasing temperature near *T*_CO_. The marked change in temperature dependence across *T*_CO_ suggests that the increase in RIXS intensity above *T*_CO_ is related to the melting of the charge order. Notably, the increase is larger at ***q***_CO_ than at other momenta. From the result in Fig. 1b,c, we can divide the increase in RIXS spectral weight above *T*_CO_ into two components. One is enhanced intensity at ***q***_CO_ and the other is momentum-independent part. Because of the coincidence of momentum and temperature, we reasonably ascribe the former component to the fluctuations of the melted charge order. To our knowledge, such momentum- and energy-resolved charge fluctuations proximate to the charge-ordered phase have never been observed experimentally thus far. On the other hand, one possibility to account for the latter component is the increase in the number of mobile holes associated with the charge order melting. In other words, holes are localized below *T*_CO_, which is indicated by a kink in resistivity^23^. Alternatively, a nuclear magnetic resonance study suggests that the number of holes in the ladder itself changes with temperature due to charge transfer between the ladder and the chain^24^.

In Sr_14−*x*_Ca_*x*_Cu_24_O_41_, the static charge order of *x* = 0 disappears suddenly on hole doping into the ladder by substituting Ca for Sr; this is considered quantum melting^18^. It is interesting to see how the charge fluctuations change with the melting. Figure 1d shows the raw RIXS spectra of *x* = 3 at 8 K and 400 K. Similar to the case of *x* = 0, enhanced intensity at ***q***_CO_ = (0, 0.2) is observed at 400 K, whereas the continuum intensity around 1 eV is almost independent of momentum at 8 K. In Fig. 1e, we plot the momentum dependence of the integrated RIXS intensity between 1.0 and 1.4 eV. Charge fluctuations are observed as enhancements at ***q***_CO_, and they are prominent at 300 K and 400 K. When holes are doped further to *x* = 6, the temperature dependence of the fluctuations changes. Figure 1f,g show the raw RIXS spectra and the momentum dependence of the integrated RIXS intensity, respectively. The enhancement at ***q***_CO_ remains, but it is rather noticeable at lower temperatures. Slight shift of the peak from ***q***_CO_ at *x* = 6 in Fig. 1g may indicate that an increase in the number of holes in the ladder causes incommensuration, which destabilizes the static charge order. Figure 1h shows a summary of the doping dependence. The radii of the circles in the figure show the magnitude of the enhancement at ***q***_CO_ which is defined as 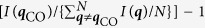, where *N* is the number of measured momentum points except for ***q***_CO_. The radius becomes zero when no enhancement is observed at ***q***_CO_. With increasing the hole concentration in the ladder, the charge fluctuations at ***q***_CO_ become prominent at lower temperatures, which means the dynamical charge correlation with five-fold periodicity become destabilized by hole doping.

Note that we extended the measurement of RIXS up to *x* = 11.5, but we could not observe the fluctuations of three-fold periodicity in the proximity of the three-fold charge ordered phase at *x* ~ 11, even though the three-fold static charge order was established by resonant elastic x-ray scattering^18^. (Detailed RIXS studies of high Ca concentrations will be published elsewhere.) These results imply that the three-fold charge order at *x* ~ 11 is qualitatively different from the five-fold charge order at *x* = 0 in the energy scale, as discussed later.

### Excitations in the triangular bilayer lattice

**Figure 2:**
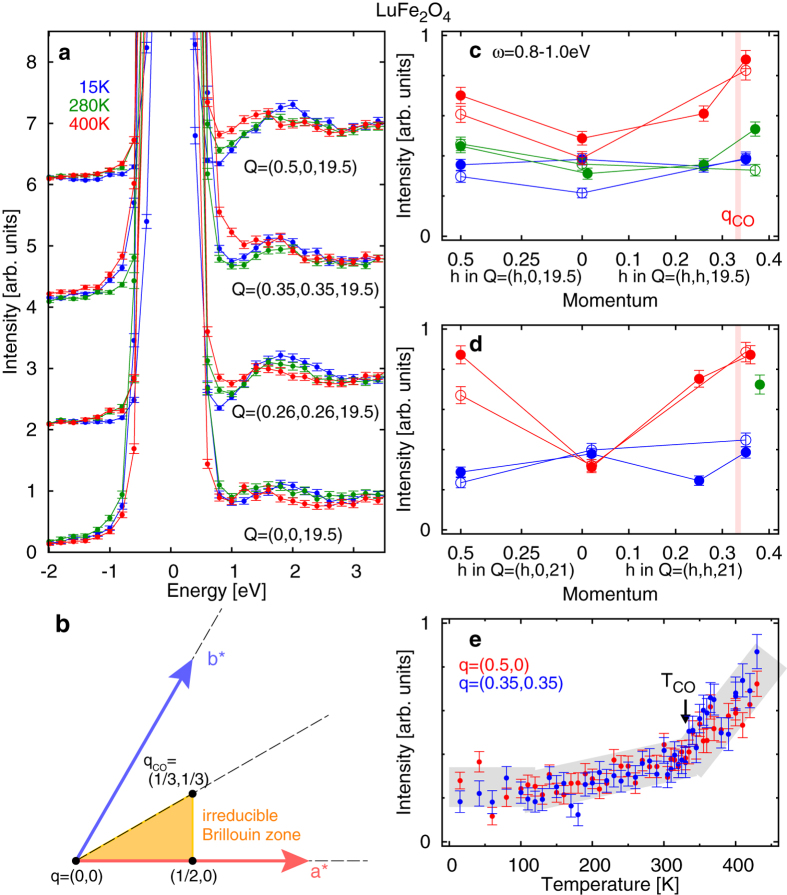
Charge excitations in the triangular-bilayer lattice in LuFe_2_O_4_. (**a**) Raw RIXS spectra measured at 15, 280 and 400 K. (**b**) Reciprocal lattice of the triangular lattice. The yellow triangle is the irreducible Brillouin zone. (**c**,**d**) Momentum dependence of integrated spectral weight at 0.8–1.0 eV. The vertical thick bars denote the in-plane propagation vector of the charge order (***q***_CO_). Open and filled circles represent the data recorded in the 1st and 2nd experiments, respectively. The two experiments were performed independently, but the same sample was measured. (**e**) Temperature dependence of integrated spectral weight at 0.8–1.0 eV. The arrow indicates the transition temperature of the charge order (*T*_CO_), and the thick line is to serve as a guide for eyes.

Since we have established the capability of RIXS to measure charge fluctuations, we proceed to the more complex case of LuFe_2_O_4_, where the charge degree of freedom is geometrically frustrated in the triangular bilayer lattice. Figure 2a shows raw RIXS spectra at the Fe *K*-edge at temperatures well below, slightly below, and above the transition temperature of three-dimensional charge order *T*_CO_ = 330 K^19^. The RIXS intensity at around 1 eV increases at high temperatures, except for the in-plane zone center ***Q*** = (0, 0, 19.5). Analogous to the result of Sr_14−*x*_Ca_*x*_Cu_24_O_41_, we ascribe the increase at around 1 eV to charge fluctuations related to the charge order. We plot the integrated RIXS intensity between 0.8 and 1.0 eV after subtracting the corresponding intensity of the anti-Stokes region (between −1.0 and −0.8 eV) in Fig. 2c,d, respectively, for ***Q*** = (*h*, *k*, 19.5) and (*h*, *k*, 21). This energy window is ascribed to the charge fluctuations between Fe^2+^ and Fe^3+^ from an optical study^25^. When the charge order is melted at 400 K, the charge fluctuations are enhanced. In distinct contrast to the case of the two-leg ladder, the enhancement above *T*_CO_ is observed not only at (close to) the propagation vector of the charge order within the layer 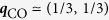 but also at other in-plane momenta 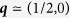 and (1/4, 1/4). Here ***q*** denotes the reduced in-plane momentum of the triangular bilayer. Temperature dependence of the integrated RIXS intensity at 0.8–1.0 eV is shown in Fig. 2e. The intensities at both 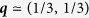 and ***q*** = (1/2, 0) begin to increase above 100 K (≪*T*_CO_), and the slope becomes steeper above *T*_CO_.

### Theoretical calculation

To confirm the qualitative difference of charge fluctuations between systems with and without geometrical frustration, we calculated the temperature dependences of the dynamical charge correlation functions (DCCF). Correlated electron systems with and without geometrical frustration are simulated using the interacting fermion models, termed the *Vt* models, on the triangular bilayer and square lattices, respectively. We have confirmed in our previous papers^20^,^26^ that this model on the triangular bilayer can reproduce the CO structure in LuFe_2_O_4_. Because the charge pattern in the ladder plane of Sr_14_Cu_24_O_41_ has not been determined experimentally, we adopt the square lattice as a model without charge frustration. A checkerboard-type CO and polar three-fold CO appear in the square and triangular bilayer lattices, respectively, at low temperatures. The DCCFs at finite temperature are calculated numerically by applying the exact diagonalization method based on the Householder algorithm to finite-sized clusters. Low-energy charge fluctuations are deduced by integrating the excitation spectra of the DCCF up to a cut-off energy as 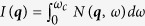, where *N*(***q***, *ω*) is the DCCF, *ω*_*c*_ is chosen to be below the insulating gap at zero temperature, and the elastic component located at around *ω* = 0, corresponding to the superlattice diffraction peak, is removed.

**Figure 3:**
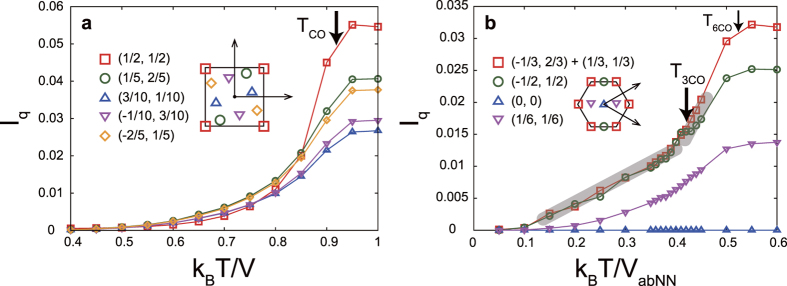
Theoretical charge fluctuation intensities. (**a**,**b**) Temperature dependences of integrated dynamical charge correlation functions in square lattice and triangular bilayer lattices. *T*_CO_ in (**a**) represents the charge-ordering temperature of checkerboard-type CO, and *T*_3CO_ and *T*_6CO_, respectively, in (**b**) represent the charge-ordering temperatures of the three-fold and six-fold COs, corresponding to three- and two-dimensional COs in LuFe_2_O_4_. Insets show the first Brillouin zones and the momenta at which the dynamical charge correlation functions were calculated.

Figure 3a,b show the temperature dependences of the integrated intensity *I*(***q***) in the square and triangular bilayer lattices, respectively. The integrated intensities are almost zero at temperatures well below *T*_CO_ in the square lattice model, implying that the static CO develops well, and begin to increase near *T*_CO_ with increasing temperature. The increase in intensity near *T*_CO_ is more prominent at the propagation vector of CO, ***q***_CO_ = (1/2, 1/2), than at other momenta. The temperature dependence of *I*(***q***) in the triangular bilayer lattice is qualitatively contrastive. There are two characteristic temperatures, termed *T*_6CO_ and *T*_3CO_ (*T*_6CO_ > *T*_3CO_), in which the six-fold and three-fold COs set in, respectively. The latter *T*_3CO_ corresponds to the experimentally observed CO temperature at *T*_CO_ = 330 K in LuFe_2_O_4_ shown in Fig. 2e. It is shown that even below *T*_3CO_, intensive low-energy charge fluctuation remains. A weak kink is confirmed at around *T*_3CO_ in the temperature dependence of *I*(***q***). These behaviors appear not only at ***q***_CO_ but also at (−1/2, 1/2).

When we focus on the temperature regions up to a few ten percent higher than *T*_CO_ and *T*_3CO_, the momentum dependence of *I*(***q***) in the triangular bilayer lattice is weaker than that in the square lattice. Note that the momenta (1/2, 0) and (−1/2, 1/2) are equivalent under the three-fold symmetry of the triangular bilayer lattice. These theoretical results explain well the experimental observations made for Sr_14_Cu_24_O_41_ and LuFe_2_O_4_.

## Discussion

For both Sr_14−*x*_Ca_*x*_Cu_24_O_41_ and LuFe_2_O_4_, charge fluctuations are observed at of the order of 1 eV. Even though the charge fluctuations may also appear at lower energy, we emphasize here that the charge fluctuations extend to large energy scale. This suggests that the strong electron-electron interaction at the same energy scale is relevant to the charge order, and we consider that the interaction at the highest-energy scale plays a primary role in the occurrence of the charge order. Other interactions, such as magnetic and electron-phonon interactions, may contribute to the charge order to some extent. Even so, energy scale of the interactions is lower than the Coulomb interaction and their role is secondary. Importance of the strong electronic interaction has been indicated in the optical studies. In the optical conductivity of Sr_14_Cu_24_O_41_, spectral weight below ~1 eV is suppressed largely with decreasing temperature, and it moves toward higher energy^27^,^28^, namely spectral transfer occurs up to the order of 1 eV, which is comparable to the charge fluctuations observed in RIXS. The large energy scale in Sr_14_Cu_24_O_41_ accords with the argument that the charge order is considered as a sort of Wigner crystallization driven by many-body electronic effects in which a detectable lattice distortion is missing^17^. In contrast, the suppression of the spectral weight in optical conductivity is significant at one order lower energy (<0.1 eV) at high Ca concentration^29^,^30^, even though a three-fold charge order is established at *x* ~ 11 by resonant elastic x-ray scattering^18^. This indicates that the charge order at *x* ~ 11 is a phenomenon at energies lower than that at *x* = 0, namely charge orders are qualitatively different between *x* = 0 and *x* ~ 11.

Similarly, in LuFe_2_O_4_, the spectral weight of optical conductivity at 0.6–1.0 eV, which is ascribed to the charge fluctuations between Fe^2+^ and Fe^3+^ in the study, is suppressed below *T*_CO_ 25, and the energy is comparable to that of the charge fluctuation observed in the present RIXS study. Therefore, the charge order in LuFe_2_O_4_ is also likely to be driven by the electron-electron interaction. The most important difference between Sr_14_Cu_24_O_41_ and LuFe_2_O_4_ is the momentum dependence of charge fluctuations. While the fluctuations are limited only at (or close to) ***q***_CO_ in the former, they are observed at two- [***q*** = (1/2, 0)], three- [***q*** = (1/3, 1/3)] and four- [***q*** = (1/4, 1/4)] periodicities in the latter. This can probably be ascribed to the geometrical charge frustration inherent in the triangular lattice. In general, difference of the free energy among various charge configurations is very small if the charge frustration exists. In the case of LuFe_2_O_4_, a theoretical calculation has demonstrated that free energies of two-, three-, and four-fold charge-ordered states are close to each other and a slight change in a parameter in the theoretical model switches the state from one to the other^20^,^26^. The three-fold charge-ordered state is realized below *T*_CO_, but, when the order is melted, competition between the states emerges as fluctuations at the finite energy.

One may concern how such charge fluctuations appear in high-*T*_c_ cuprates because the topic of charge-ordered states in underdoped regions has recently attracted great interest as a competing phenomenon to superconductivity^31^,^32^,^33^. Some of the present authors observed enhancement of the intraband excitations at the propagation vector of the charge order ***q***_CO_ in a Cu *K*-edge RIXS study of the charge-stripe-ordered La_2−*x*_(Ba,Sr)_*x*_CuO_4_ (*x* ~ 1/8)^34^. The result of the high-*T*_c_ cuprates is similar to the present study of Sr_14−*x*_Ca_*x*_Cu_24_O_41_ in the sense that the intraband excitations around 1 eV form a peak at ***q***_CO_ in the momentum space, but the enhancement is qualitatively different between the two systems. In the high-*T*_c_ cuprates, the enhancement was observed in the ordered phase and thereby collective stripe excitations and anomalous softening of the charge excitonic modes of the in-gap states are proposed as a possible origin. We note that a subsequent study of the high-*T*_c_ cuprates^35^ showed that the enhancement near ***q***_CO_ extends to the overdoped region, indicating that direct relation of the enhancement to the charge-stripe order is unlikely. In contrast, the enhancement in Sr_14−*x*_Ca_*x*_Cu_24_O_41_ is prominent when the order is melted in the disorderd phase, and it is less clear or missing in the ordered phase. Recently, Cu *L*_3_-edge RIXS was also applied to the study of charge-ordered cuprates. Though a superlattice peak of the charge order was observed at the elastic or quasi-elastic position in the ordered phase^31^,^36^,^37^ and magnetic excitations might change slightly across the ***q***_CO_^36^,^38^, charge fluctuations at a finite energy have not been observed in the disorded phase so far. Observation of the charge fluctuations in the high-*T*_c_ cuprates still remains to be done. Difficulty of the observation may come from energy scale of the charge fluctuations lower that the energy resolution. Judging from the temperature dependence of optical conductivity of the stripe-ordered high-*T*_c_ cuprates^39^,^40^, charge fluctuation associated with the thermal melting of the charge order would appear at ~0.1 eV in the high-*T*_c_ cuprates. If so, observation of the charge fluctuation is a challenging subject even in the state-of-the-art RIXS spectrometer. In Sr_14_Cu_24_O_41_, low dimensionality of the two-leg ladder and low electric conductivity prevent carriers from screening the electron-electron interaction, which keeps the charge fluctuation at higher energies. The substitution of Ca for Sr makes the compound conductive not only along the leg but also along the rung in Sr_14−*x*_Ca_*x*_Cu_24_O_41_ 41; consequently, the screening is effective at high Ca concentrations, as is in the high-*T*_c_ cuprates.

In the present study, we show that charge fluctuations proximate to charge order appear in the RIXS spectra of Sr_14−*x*_Ca_*x*_Cu_24_O_41_ and LuFe_2_O_4_. Because the charge fluctuations resolved in both energy and momentum have never been observed so far, our RIXS results unfold a new aspect of charge fluctuations in strongly correlated electron systems.

## Methods

We prepared single crystals of Sr_14−*x*_Ca_*x*_Cu_24_O_41_ (*x* = 0, 3 and 6) and LuFe_2_O_4_ by the traveling solvent floating zone method. The sample surfaces normal to the two-leg ladder plane (*ac*-plane) and the triangular lattice plane (*ab*-plane), respectively, in Sr_14−*x*_Ca_*x*_Cu_24_O_41_ and LuFe_2_O_4_ were irradiated with x-rays.

The Cu and Fe *K*-edge RIXS experiments were performed at BL11XU in SPring-8. Incident x-rays were monochromatized by a Si(111) double-crystal monochromator, and the energy bandwidth was reduced further by a Si(400) channel-cut monochromator for both edges.

At the Cu *K*-edge, horizontally scattered x rays were analyzed in energy by a spherical Ge(733) analyzer, and the total energy resolution estimated from the full width at half maximum (FWHM) of the elastic peak was approximately 400 meV. Single crystals of Sr_14−*x*_Ca_*x*_Cu_24_O_41_ were mounted such that the *bc*-plane was parallel to the scattering plane when momentum transfer along the a^*^-direction was zero. Under this experimental geometry, the polarization of the incident photons (***ε***_i_) has almost equal *b* and *c* components. The b^*^ component of momentum transfer was selected such that the scattering angle (2*θ*) was close to 90°. Then, absolute momentum transfers (***Q***) for *x* = 0, 3 and 6 were (*H*, 13.6, *L*), (*H*, 13.4, *L*) and (*H*, 13.2, *L*), respectively. Incident photon energy was fixed at 8993 eV for the RIXS measurements, the same as in a previous study^22^. This photon energy is slightly higher than the poorly-screened final state of the x-ray absorption of ***ε*** ‖ ***b***, where ***ε*** denotes the photon polarization in x-ray absorption. This incident photon energy is close to the energy where a core-hole is created at a hole-doped site, and it was demonstrated that the Cu *K*-edge RIXS spectra agree well with the dynamical charge correlation function when the incident photon energy is tuned to the condition^35^,^42^.

In Fig. 1b,e,g, we integrated the intensity between 1.0 and 1.4 eV. More precisely, we added the intensity of three data points at 1.0, 1.2 and 1.4 eV because we measured the spectra at 0.2 eV step. In order to show how the results are robust irrespective of the integration window, we plot the intensity of each data point after subtracting the corresponding intensity of the anti-Stokes region in Supplementary Fig. S1. The enhancement at **q**_co_ is still clear. Furthermore, we measured the key spectra (8 K and 400 K for x = 0, and 8 K for x = 6) twice and the second scans are shown in Supplementary Fig. S2. They are almost identical to the spectra in Fig. 1(a,f).

We used a Ge(620) analyzer for the Fe *K*-edge. Total energy resolution was approximately 500 meV. The horizontal scattering plane was spanned along the 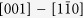 directions. The incident photon energy was set to 7130 eV, where excitations at a few eV were enhanced resonantly, as shown in Supplementary Fig. S3.

Theoretical calculations were performed on the interacting fermion systems modeled by the spinless fermion *Vt*-model Hamiltonian given by


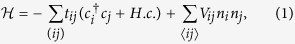


where 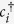 (*c*_*i*_) is the creation (annihilation) operator for a spinless Fermion at site *i* and 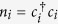 is a fermion number operator. Inter-site fermion hoppings (*t*_*ij*_) and Coulomb interactions (*V*_*ij*_), respectively, were considered up to the 2nd and the 3rd neighboring sites in a triangular bilayer lattice. In a square lattice, both *t*_*ij*_ and *V*_*ij*_ were introduced between the nearest neighbor sites. The cluster mean-field method was applied to finite-sized clusters. We used the open boundary condition, in which the Coulomb interaction term (the second term in Eq. (1)) is decoupled, and introduced the mean fields 〈*n*_*i*_〉. The Hamiltonians of finite-sized systems were analyzed by the Householder algorithm, and the mean-fields were determined self-consistently with the states in a cluster. Cluster sizes were taken as 10 and 12 for square and triangular bilayer lattices, respectively. We calculated numerically the dynamical charge correlation function at a finite temperature defined by


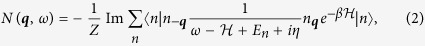


where *Z* is the partition function, *β* is the inverse temperature and *η* is a small numerical constant.

## Additional Information

**How to cite this article**: Yoshida, M. *et al.* Observation of momentum-resolved charge fluctuations proximate to the charge-order phase using resonant inelastic x-ray scattering. *Sci. Rep.* **6**, 23611; doi: 10.1038/srep23611 (2016).

## Supplementary Material

Supplementary Information
