# Supplementary material for: Observation of momentum-resolved charge fluctuations proximate to the charge-order phase using resonant inelastic x-ray scattering
Source: Sci Rep. 2016 Mar 29;6:23611. doi: 10.1038/srep23611 (PMC4817204; doi:10.1038/srep23611)
Supplement: Supplementary Information [file srep23611-s1.pdf]

Supplementary information of Observation of momentum-resolved charge fluctuations proximate to the charge-order phase using resonant inelastic x-ray scattering

M. Yoshida[1,2], K. Ishii[1,*], M. Naka[3], S. Ishihara[2,4], I. Jarrige[1,+], K. Ikeuchi[1,5], Y. Murakami[6], K. Kudo[7], Y. Koike[8], T. Nagata[7], Y. Fukada[7], N. Ikeda[7], and J. Mizuki[1,9]

[1]SPring-8, Japan Atomic Energy Agency, Sayo, Hyogo 679-5148, Japan
[2]Department of Physics, Graduate School of Science, Tohoku University, Sendai 980-8578, Japan
[3]RIKEN Center for Emergent Matter Science (CEMS), Wako 351-0198, Japan
[4]CREST, JST, Chiyoda, Tokyo 102-0076, Japan
[5]Research Center for Neutron Science and Technology, Comprehensive Research Organization for Science and Society (CROSS), Tokai, Ibaraki 319-1106, Japan
[6]Institute of Materials Structure Science, High Energy Accelerator Research Organization, Tsukuba, Ibaraki 305-0801, Japan
[7]Department of Physics, Okayama University, Okayama 700-8530, Japan
[8]Department of Applied Physics, Graduate School of Engineering, Tohoku University, Sendai 980-8579, Japan
[9]School of Science and Technology, Kwansei Gakuin University, Sanda, Hyogo 669-1337, Japan
[*]kenji@spring8.or.jp
[+]Present address: Photon Sciences Directorate, Brookhaven National Laboratory, Upton, New York 11973, USA

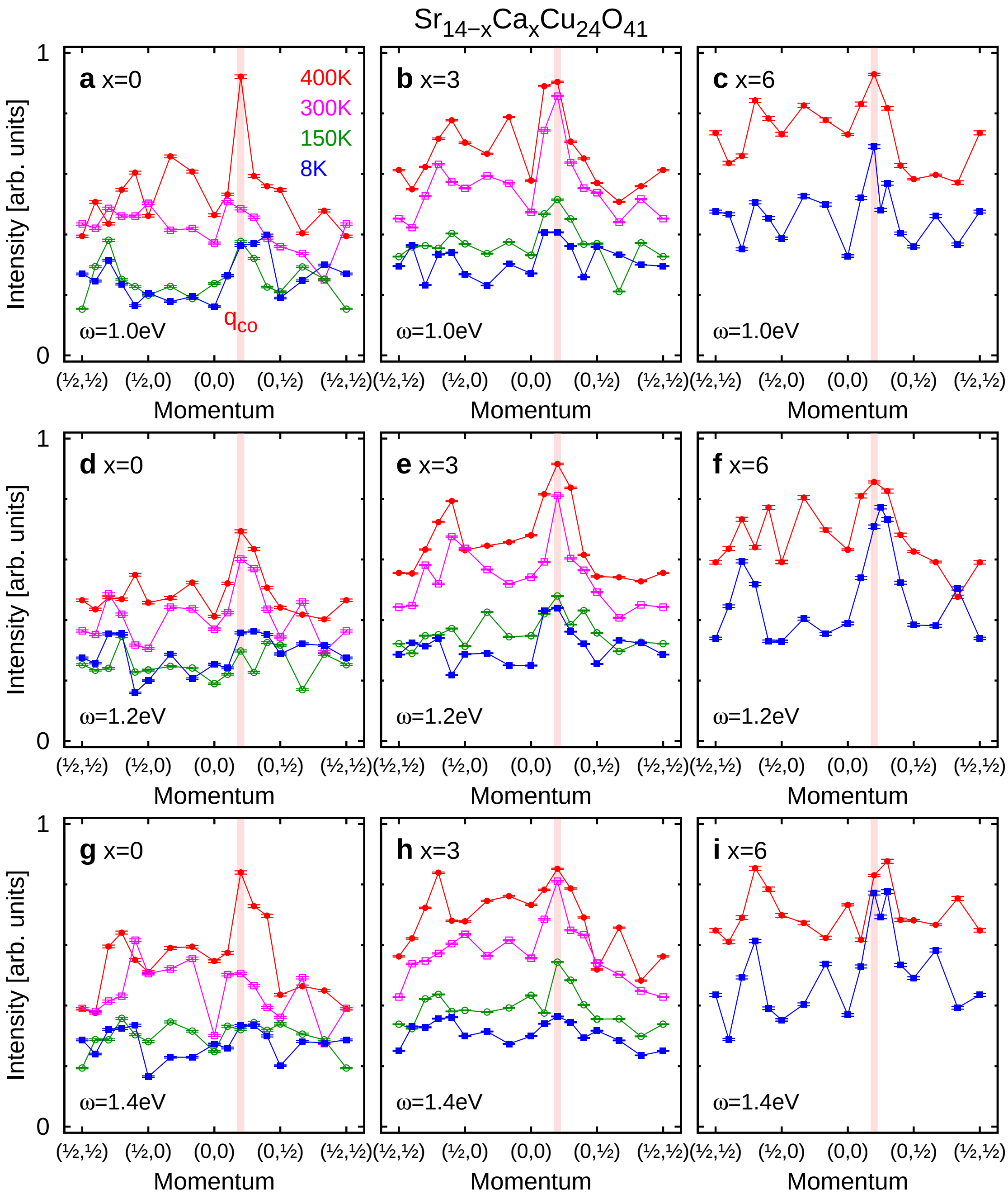

Figure S1. **Momentum dependence of RIXS intensityin $Sr_{14-x}Ca_xCu_{24}O_{41}$.** RIXS spectral intensity at 1.0, 1.2, and 1.4 eV are plotted as a function of momentum. The vertical thick bars denote the propagation vector of the charge order at $x = 0$ ($\boldsymbol{q}_{\mathrm{CO}}$).

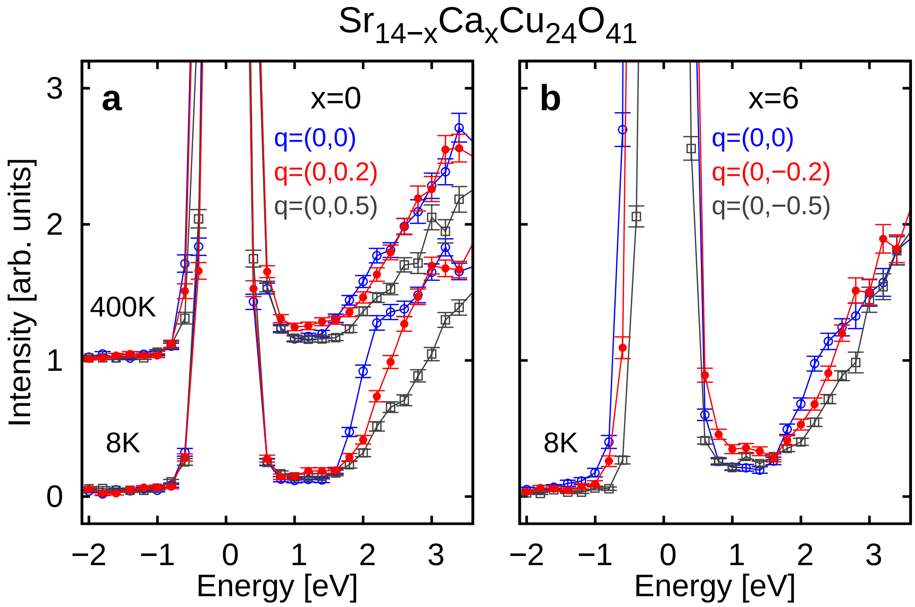

Figure S2. **Repeated scans of RIXS spectra on $Sr_{14-x}Ca_xCu_{24}O_{41}$.**

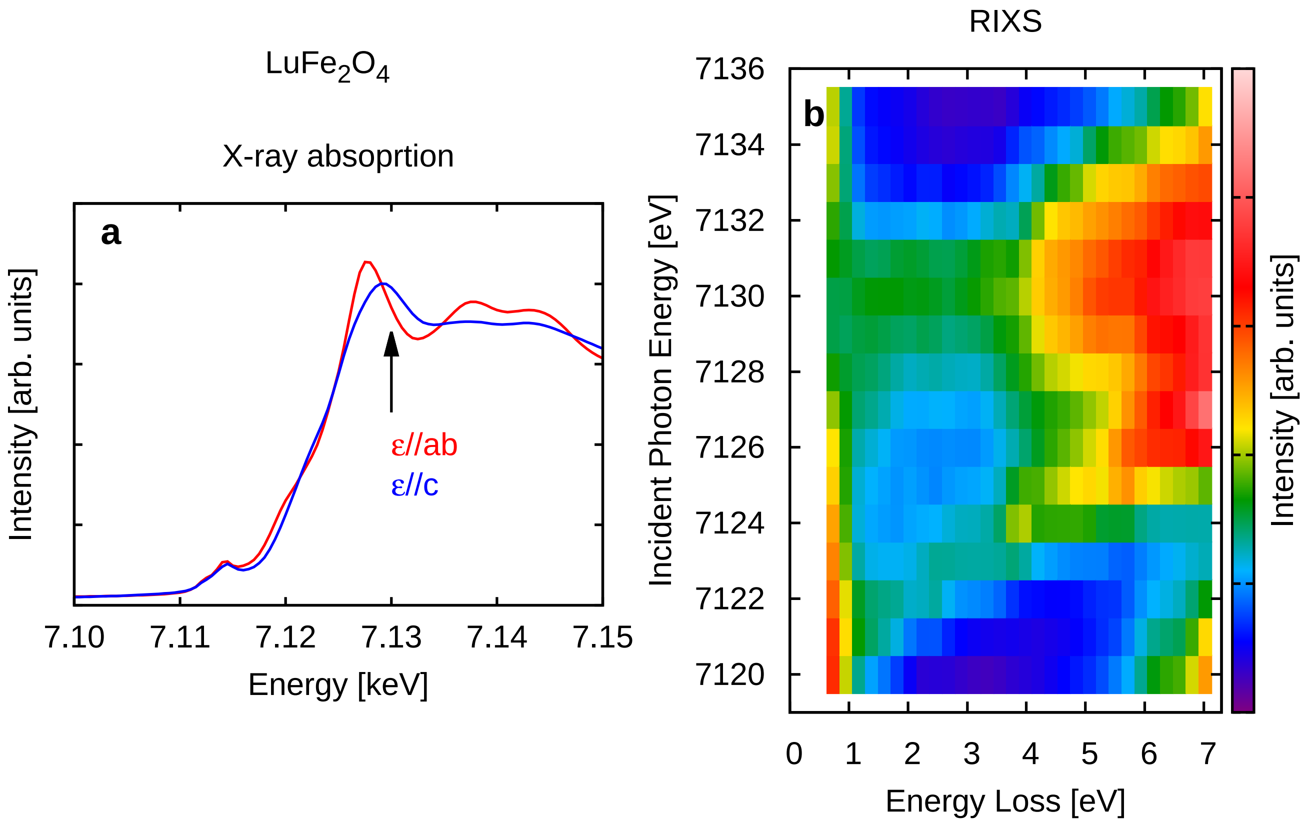

Figure S3. X-ray absorption spectra and incident photon energy dependence of RIXS of $LuFe_2O_4$ at the Fe *K*-edge. **(a)** Polarization-dependent x-ray absorption spectra. The arrow indicates the incident photon energy for the RIXS measurement in the main text. **(b)** Incident photon energy dependence measured at **Q** = (0,0,19).
